# Mental health and creativity in university students: a multidimensional mediation model of cognition, emotion, and motivation

**DOI:** 10.3389/fpsyg.2025.1647823

**Published:** 2025-11-11

**Authors:** Shengguo Zhu, Xinchen Leng

**Affiliations:** 1 Department of Fundamental Courses, Hunan Police Academy, Changsha, China; 2 Department of Business, Monash University, Melbourne, VIC, Australia

**Keywords:** mental health, creativity, college students, creative cognitive ability and executive function, intrinsic motivation and achievement goal orientation, emotions

## Abstract

Mental health is a fundamental basis for the comprehensive development of college students, and creativity is a key factor in academic success and future adaptability. Although previous studies have suggested a close association between the two, the mechanisms linking them remain insufficiently clarified. This study used a cross-sectional design with a sample of 600 Chinese college students, who completed online questionnaires assessing mental health, creativity, and related psychological factors. Descriptive statistics, correlation and regression analyses, and mediation tests were conducted. The findings showed that mental health significantly and positively predicted creativity, indicating that students with better mental health reported higher creativity. Further analysis revealed that cognitive pathways played a partial mediating role, underscoring the importance of cognitive flexibility in the development of creativity, while the mediating roles of emotional and motivational pathways were not significant, suggesting that their influence may be context-dependent. These results demonstrate that mental health enhances creativity both directly and indirectly through cognitive processes, providing theoretical support for cognitive-based models of creativity and offering practical implications for integrating cognitive training and mental health promotion into educational practice. Future research should adopt longitudinal designs and diverse methodological approaches to capture the dynamic interplay between mental health and creativity.

## 1 Introduction

Creativity is defined as the ability to produce novel and adaptive ideas (Guilford, 1950), and is the cornerstone of social progress and individual problem-solving. From driving technological innovation (Hennessey and Amabile, 2010) to enhancing an individual’s resilience in the process of life transformation (Runco, 2014), the meaning of creativity spans many fields. Empirical evidence suggests that creativity has always been associated with improved workplace adaptability (Amabile and Pratt, 2016) and effective coping strategies in an educational environment (Beghetto and Kaufman, 2007). Adolescence is a crucial period for the development of creativity, which is not only related to an individual’s academic performance and personal growth, but also directly affects their future career development and social adaptability (Sternberg and Lubart, 1996; Beghetto and Kaufman, 2007).

Many studies have highlighted the complex relationship between mental health and creative output. Some studies suggest that subclinical mood disorders may temporarily enhance divergent thinking (Kyaga et al., 2013), but meta-analysis results show that long-term mental distress often undermines the cognitive flexibility necessary for creative output (Byron et al., 2010). It is notable that Fredrickson (2001) “broadening and constructing” theory suggests that positive emotions broaden attention and thereby promote creativity. In contrast, severe anxiety is associated with persistent thinking patterns that limit the exertion of creative potential (Beaty et al., 2014). Mental health problems among adolescents have become increasingly prominent in recent years, such as negative emotions like anxiety, depression and stress, which have had a wide-ranging impact on the growth of the adolescent population (Twenge et al., 2019). Therefore, exploring how mental health affects the development of adolescent creativity not only helps to gain a deeper understanding of the underlying mechanisms of creativity development, but also has significant practical implications for promoting mental health and fostering creativity among adolescents.

Studies have found a significant link between mental health status and creativity, but the underlying mechanisms and specific pathways of their relationship have not been systematically and clearly explained (Kaufman and Beghetto, 2009; Ivcevic and Brackett, 2015). On the one hand, positive mental health status may promote cognitive flexibility (Diamond, 2013), enhance executive function (Zabelina and Robinson, 2010), and improve emotional regulation ability (Fredrickson, 2001; Davis, 2009) and strengthening intrinsic motivation (Amabile, 2012) can effectively enhance adolescent creativity performance; On the other hand, negative mental health conditions such as anxiety and depression may suppress or distort the development of creative thinking (Silvia and Kaufman, 2010; Byron and Khazanchi, 2011). Therefore, clarifying the specific mechanisms and pathways by which mental health affects adolescent creativity is crucial for both theoretical research and practical intervention.

Based on the integration of existing theoretical frameworks and empirical research, this study selects cognition, emotion, and motivation as mediating variables to systematically examine their role in the relationship between mental health and creativity. First, from a cognitive perspective, mental health status (such as anxiety, depression, or positive emotions) may change an individual’s creative performance by influencing executive functions (such as working memory, cognitive flexibility) and divergent thinking (Diamond, 2013; Zabelina and Robinson, 2010). Secondly, the theoretical basis of the emotional path stems from Fredrickson (2001) extender-construction theory, which states that positive emotions can broaden cognitive resources and enhance mental flexibility, while negative emotions may suppress creativity by narrowing attention (Byron et al., 2010). Finally, the choice of motivational paths is based on self-determination theory (Deci and Ryan, 2000), and mental health may be achieved by regulating intrinsic motivation (such as interests, autonomy) and achievement goal orientation mastering goals vs. indirectly, it affects the persistence of an individual’s engagement in creative activities (Amabile, 2012). These three pathways together form a multi-dimensional mechanism model that explains both the direct promoting effect of mental health on creativity and the potential boundaries of its indirect effects, such as the interference of mood regulation strategies or extrinsic motivations. Therefore, this study aims to provide a more detailed theoretical explanation and practical intervention target for the complex relationship between mental health and creativity by examining these three mediating pathways.

This study aims to systematically review and analyze relevant research results at home and abroad in recent years, specifically explore the mechanism of the effect of mental health on adolescent creativity, clarify the three specific pathways of cognition, emotion and motivation, and propose potential directions and methodological suggestions for future research, with the expectation of providing theoretical support and practical reference for the cultivation of adolescent creativity and the intervention of mental health.

## 2 Literature review

### 2.1 Creativity

Creativity is generally defined as an individual’s ability to produce ideas or products that are novel and have practical value (Runco and Jaeger, 2012). For a long time, there have been many different opinions about the meaning and nature of creativity, but it is generally accepted that creativity should have both “novelty” and “appropriateness” or “value” (appropriateness). That is, being able to solve practical problems in a specific context or social context (Amabile, 2012; Runco and Jaeger, 2012). Teen creativity, on the other hand, emphasizes the creative potential and achievements shown by individuals in their daily learning and life, specifically in problem-solving, critical thinking, innovative consciousness and the production of creative products (Kaufman and Beghetto, 2009).

The measurement paradigm of creativity mainly consists of three levels: creative cognitive processes, creative personality traits, and creative output. Specifically, creative cognition is usually measured by divergent thinking tests (such as the Torrance Creative Thinking Test, TTCT), focusing on indicators such as the fluency, flexibility, and uniqueness of an individual’s thinking; Creative personality, on the other hand, focuses on stable traits such as openness, self-confidence, and curiosity, and is usually measured by questionnaire self-assessment methods; Creative output is more likely to be measured by expert subjective evaluation methods, such as the Consensual Assessment Technique (CAT), where experts score works such as paintings, essays, and designs created by teenagers. To assess their actual creative performance (Amabile, 1983; Runco and Jaeger, 2012). During adolescence, research often tends to combine multiple measurement methods to provide a more comprehensive reflection of the underlying development of creativity.

### 2.2 Students’ mental health

Mental health is a positive, stable and well-adapted state of mind. The World Health Organization defines mental health as an individual’s ability to successfully realize their potential, effectively cope with normal stress in life, and contribute to society efficiently in both work and life (World Health Organization, 2018). Due to the rapid changes in physical, psychological and social roles during adolescence, mental health is characterized by phases, sensitivity and instability. Therefore, adolescent mental health is not merely the absence of mental illness, but rather the ability of an individual to effectively regulate emotions, adapt to the environment, build a positive self-identity, and interact harmoniously with society (Keyes, 2002).

Studies have shown that adolescent mental health can generally be divided into several core dimensions: the first is the emotional health dimension, which is reflected in the dynamic balance between positive and negative emotions; The second is the cognitive dimension, which includes individual cognitive flexibility, attention regulation and executive function levels; The third is the social adaptation dimension, which reflects an individual’s ability to adapt and interact with people in different situations such as family, school, and society (Keyes, 2002; Suldo et al., 2011). In addition, the dimension of resilience, which refers to an individual’s ability to quickly restore psychological balance after experiencing stress, setbacks, or even traumatic events, is reflected in good stress coping and emotional regulation abilities (Masten and Barnes, 2018). Combining these dimensions, adolescent mental health is not only related to an individual’s daily emotional experience and social adaptation, but may also directly or indirectly affect the development of cognitive functions in adolescents, thereby further influencing creativity.

### 2.3 Impact of mental health on students’ creativity

#### 2.3.1 A positive psychology perspective

Positive psychology emphasizes how an individual’s positive emotions and mental health status contribute to their cognition, emotions, and behaviors. Fredrickson (2001) “broad-and-build Theory” suggests that positive emotions can expand an individual’s range of attention, thinking and acting patterns, thereby helping the individual to build more flexible cognitive structures and resources. It effectively promotes the development of creative thinking and the improvement of problem-solving skills. In addition, positive mental health conditions (such as high psychological resilience and high emotional regulation ability) enable individuals to maintain an open mindset and a positive willingness to explore even in stressful situations, thereby continuously exerting their creative potential (Zabelina and Robinson, 2010; Masten, 2018).

Classic theories in the field of creativity, such as the “Componential Theory of Creativity” proposed by Amabile (1996), also point out that creative activities are closely related to an individual’s intrinsic motivation. Individuals with high levels of mental health tend to have stronger intrinsic motivation and self-efficacy, and this positive psychological experience drives individuals to engage more deeply and consistently in creative activities (Amabile, 1996; Kaufman and Beghetto, 2009). Therefore, from the perspective of positive psychology, the promoting effect of mental health on creativity can be reflected through mediating variables such as positive emotions, intrinsic motivation, and cognitive flexibility. At the same time, Resilience Theory, as one of the important theoretical foundations of positive psychology, is also of great value for understanding the relationship between adolescent mental health and creativity. Resilience is often defined as an individual’s ability to adapt positively in adverse or stressful situations, including the ability to manage emotions effectively, the flexible cognitive ability to cope with stress, and the ability to maintain or restore a positive mental state (Masten and Barnes, 2018). Studies have shown that adolescents with high psychological resilience are better at coping with environmental stress and negative emotions, and exhibit higher creative thinking in challenging situations (Masten, 2018). Furthermore, the cognitive theory perspective highlights how mental health affects an individual’s cognitive function and thereby influences the development of creativity. Diamond (2013) points out that the executive functions of adolescent individuals, including working memory, cognitive flexibility, and inhibitory control, are largely influenced by mental health status. Negative emotions such as anxiety and depression may impair executive function and cognitive flexibility in adolescents, thereby limiting the performance of individual creative thinking (Byron and Khazanchi, 2011); On the contrary, a positive mental state improves the efficiency of executive function, thereby enhancing the flexibility and originality of creative thinking (Zabelina and Robinson, 2010; Diamond, 2013).

It can be seen from this that the extension of positive psychology - the constructive theory, the creativity component theory, and the psychological resilience theory and cognitive theory together form the theoretical basis for understanding the impact of mental health on adolescent creativity. These theories lay a solid foundation for in-depth exploration of specific mechanisms of action, such as cognitive, emotional, and motivational pathways.

#### 2.3.2 Cognitive pathways

From the perspective of cognitive pathways, the mechanism by which mental health affects adolescent creativity is mainly reflected in the moderating and promoting effects of mental health factors on individual cognitive functions. Specifically, mental health status may affect the development of creativity directly or indirectly by influencing an individual’s executive function, cognitive flexibility, and cognitive control ability (Zabelina and Robinson, 2010; Diamond, 2013).

First of all, Executive Functions are important cognitive foundations of creative thinking, including core components such as cognitive inhibition, working memory, and cognitive flexibility. Diamond (2013) pointed out that the mental health status of adolescents significantly affects the development and functioning of their executive functions. For example, adolescents who have been in a state of anxiety or depression for a long time often have weakened working memory ability and have difficulty flexibly mobilizing and managing cognitive resources. This inhibits the ability to solve creative problems (Byron and Khazanchi, 2011). In contrast, adolescents with better mental health performed better in executive function. They were able to organize and manage information more effectively and showed greater cognitive flexibility and divergent thinking in creative tasks (Diamond, 2013). Secondly, psychological resilience, as a key protective factor for mental health, also has a positive impact on creative development by influencing cognitive patterns. Individuals with high psychological resilience tend to reconstruct challenging situations as manageable, opportunistic events, thereby enhancing resilience to stress and cognitive flexibility (Masten and Barnes, 2018). Masten (2014) study shows that adolescents with strong psychological resilience are better at quickly adjusting cognitive strategies and flexibly changing problem-solving approaches when encountering challenging problems, which helps maintain higher creative performance. Therefore, psychological resilience plays an important mediating role in the relationship between mental health and creativity by optimizing an individual’s cognitive patterns and executive functions. In addition, some empirical studies have found that moderate psychological stress can improve cognitive flexibility and creative performance to a certain extent, showing an “inverted U-shaped” stress-creativity relationship (Byron et al., 2010). In this context, mental health acts as a “cognitive buffer,” meaning that individuals with good mental health are better able to cope with and utilize the cognitive activation effect of stressful situations, thereby increasing the likelihood of creative performance (Byron et al., 2010).

In summary, the cognitive pathway clearly reveals how mental health factors work together on adolescent creativity development through cognitive mechanisms such as executive function, cognitive flexibility, emotional regulation ability, and psychological resilience. This understanding not only contributes to the in-depth analysis of the relationship between mental health and creativity at the theoretical level, but also provides specific psychological interventions and educational strategies for the development of adolescent creativity in practice (Zabelina and Robinson, 2010; Diamond, 2013; Ivcevic and Brackett, 2015).

#### 2.3.3 Emotional pathways

Emotional pathways emphasize the direct or indirect impact of mental health on an individual’s creative thinking through positive and negative emotions. Emotions are not only an immediate psychological response, but also profoundly affect an individual’s creative performance by regulating the allocation of cognitive resources and changes in thinking patterns (Ashby et al., 1999; Fredrickson, 2001; Gross, 2002).

Positive emotions play a significant role in promoting creativity. Fredrickson (2001) broad-and-build Theory clearly states that positive emotions such as happiness, satisfaction, and interest can Broaden an individual’s range of attention and increase the availability of cognitive resources. This helps individuals build broad and flexible cognitive structures (Fredrickson and Branigan, 2005). The neuropsychological research by Ashby et al. (1999) also supports this theory, suggesting that positive emotions activate the dopamine system in the brain, thereby improving an individual’s cognitive flexibility and problem-solving ability and directly promoting creative performance. Isen (1999) study also confirmed that the experience of positive emotions helps to improve an individual’s problem-solving performance in complex tasks, as this makes it easier for the individual to explore new information and methods. Among adolescents, positive emotional experiences are particularly effective in promoting creativity. Tugade and Fredrickson (2004) found that adolescents with high psychological resilience are better at using positive emotions to recover from negative events. They not only have better emotional regulation abilities, but also are more flexible in responding to challenges and show higher creativity. This suggests that developing teenagers’ experiences of positive emotions and their ability to effectively regulate positive emotions may be one of the key ways to promote the development of teenagers’ creativity.

In contrast, negative emotions such as anxiety, depression and stress have a more complex impact on creativity. A meta-analysis by Byron et al. (2010) suggests that negative emotions can have very different effects on creativity in different situations. High intensity of negative emotions is often shown to limit the breadth of attention and reduce the effective use of individual cognitive resources, thereby leading to a decline in creativity (Byron et al., 2010). However, moderate negative emotions such as mild anxiety or frustration may stimulate an individual’s awareness of active exploration, increase sensitivity to problems, and thereby promote creative performance (George and Zhou, 2002). Kaufmann and Vosburg (1997) also proposed the “moderate stress promotes creativity” effect, emphasizing that negative emotions in specific situations can activate an individual’s alertness and enable them to come up with more innovative solutions. In addition, emotion regulation strategies play a significant role in the emotional pathway. Gross (2002) points out that emotional regulation strategies, such as Cognitive Reappraisal and Expressive Suppression, have significantly different effects on the development of an individual’s creativity. Among them, cognitive reappraisal can effectively reduce the damage of negative emotions to cognitive function, enhance cognitive flexibility and openness, and thereby indirectly promote the development of creativity. Tugade and Fredrickson (2004) also found that individuals with higher psychological resilience tend to use positive emotion-regulating strategies to restore positive emotional states and thus exert creativity more effectively. From a neuropsychological perspective, the neuropsychological theory of positive emotions proposed by Ashby et al. (1999) suggests that positive emotions may enhance cognitive flexibility and innovative problem-solving abilities by influencing levels of neurotransmitters in the brain, such as dopamine, providing solid neuroscientific evidence for the emotional pathway.

The emotional pathways reveal how mental health affects creativity in adolescents through emotional experiences and emotional regulation strategies. Positive emotions often have an expansive effect, enhancing cognitive flexibility and creative thinking, while the impact of negative emotions depends on emotional intensity and individual regulatory strategies. Therefore, emphasizing positive intervention in the emotional state of adolescents in practice and the development of emotional regulation strategies will provide crucial support for the effective development of creativity.

### 2.4 Motivation theory

Motivation is another important path through which mental health influences the development of creativity in adolescents. According to motivation theory, an individual’s level of intrinsic motivation, self-efficacy, and orientation toward achievement goals in the face of a task or challenge significantly affect their creative performance (Bandura, 1997; Deci and Ryan, 2000; Elliot and McGregor, 2001). This section will specifically explore how mental health affects adolescent creativity development through the motivational path from three aspects: intrinsic motivation, self-efficacy, and achievement goal orientation.

Intrinsic motivation is regarded as one of the core driving forces of creativity development. The Self-Determination Theory (SDT) suggests that when individuals possess higher levels of intrinsic motivation, they are more inclined to actively explore and solve problems, thereby demonstrating stronger creative thinking and innovative abilities (Deci and Ryan, 2000). Amabile (1983) classic study further confirmed that intrinsic motivation is a key factor in adolescents’ creativity development, where interest and curiosity-driven engagement can significantly enhance creative output. The “Flow Theory” proposed by Czikszentmihalyi (1990) also supports this view: when adolescents engage in activities they are genuinely interested in, they are more likely to enter a state of flow and display higher levels of creative thinking. Thus, adolescents with better mental health are often more capable of experiencing and sustaining intrinsic motivation, which in turn enhances creative performance.

Beyond intrinsic motivation, self-efficacy also plays an important role in creativity development. Self-efficacy refers to an individual’s belief in their ability to successfully complete a task or achieve a goal (Bandura, 1997). Research has shown that adolescents with high self-efficacy are more willing to take on challenges and remain persistent in the face of difficult tasks, leading to higher levels of creativity (Schunk, 1985; Zimmerman, 2000a). From the perspective of mental health, positive psychological states help maintain stable self-efficacy, enabling adolescents to face problems and setbacks with confidence and perseverance, thereby increasing the likelihood of creative attempts (Bandura, 1997). In contrast, depression or anxiety often undermines self-efficacy, resulting in task avoidance and reduced creative performance.

Building on this, achievement goal orientation provides another perspective for understanding adolescent creativity. Achievement goal theory distinguishes between mastery goals and performance goals (Elliot and McGregor, 2001; Dweck and Leggett, 1988). Adolescents oriented toward mastery goals focus on improving their abilities and skills, are more willing to embrace challenges, and tend to explore new approaches, all of which are conducive to creative development. In contrast, adolescents oriented toward performance goals are more concerned with external evaluation and competition outcomes, which may lead them to avoid creative activities due to fear of failure (Dweck and Leggett, 1988). Harter’s (1978) theory of effectance motivation also indicates that when adolescents are motivated to seek competence improvement rather than avoid failure, they generally exhibit higher levels of creative thinking. Adolescents with better mental health are more likely to adopt mastery goal orientations, actively responding to challenges and enhancing creativity, whereas those with poorer mental health are more prone to adopt avoidance goals, thus limiting further creative development (Ryan and Deci, 2000). Based on the above research findings, the motivational path provides important theoretical and empirical support for the relationship between mental health and adolescent creativity. Mental health status dynamically affects the development of creativity by influencing the level of intrinsic motivation, self-efficacy, and achievement goal orientation of adolescents. Therefore, in the practice of mental health intervention and creativity development, particular emphasis should be placed on enhancing the intrinsic motivation, self-efficacy and goal orientation of adolescents to promote the maximization of their creative potential.

### 2.5 Emotion-cognitive theory perspective

The close and complex interaction between emotions and cognition is regarded as one of the important perspectives for understanding how mental health affects creativity (Fredrickson, 2001; Ivcevic and Brackett, 2015). According to the emotion-cognitive theory, an individual’s emotional state significantly affects their cognitive processes, including attention, memory, problem-solving and decision-making (Zabelina and Robinson, 2010; Diamond, 2013). Especially during adolescence, a crucial period of rapid psychological development, changes in emotional states can have significant and long-term effects on one’s creative cognition and performance.

Positive emotions are widely recognized for their role in promoting creativity. Positive emotions can significantly expand an individual’s cognitive resources, making it easier for the individual to enter a state of cognitive openness, thereby enhancing divergent thinking, cognitive flexibility, and the ability to solve problems innovatively. This is because positive emotional states can expand an individual’s range of attention, increase the availability of attention resources, make it easier for the individual to associate with novel and diverse information, and help improve the fluency and uniqueness of creative thinking (Fredrickson and Branigan, 2005). In contrast, the impact of negative emotions on creativity is more complex. Some studies have shown that negative emotions such as anxiety and depression often limit the allocation of cognitive resources in teenagers, resulting in a narrow range of attention and an excessive focus on the problem itself, thereby reducing divergent thinking and creative performance (Byron and Khazanchi, 2011). However, some studies have found that moderate levels of negative emotions, such as appropriate anxiety, may stimulate higher levels of creativity in certain situations. This phenomenon is known as the “negative mode-creativity facilitation effect” (George and Zhou, 2002). Therefore, the academic community still needs to further clarify the specific cognitive mechanisms and boundary conditions of different emotional types, intensities, and their effects on creativity. In recent years, the ability to regulate emotions has gradually become an important perspective for understanding the development of creativity among teenagers. Ivcevic and Brackett (2015) pointed out that adolescents with high emotional regulation ability not only manage their own emotional experiences effectively, but also actively choose emotional regulation strategies that help enhance creativity (such as cognitive reassessment) to avoid the negative impact of negative emotions on cognitive function. This ability to actively regulate emotions and flexibly mobilize cognitive resources is regarded as an important protective factor for creativity. In addition, the ability to regulate emotions may indirectly promote the development of creative thinking by influencing an individual’s attention control and cognitive flexibility (Zabelina and Robinson, 2010). In addition, the interaction between emotion and cognition also involves neural mechanisms in the brain. Recent neuroscience studies have shown that changes in emotional states may directly affect functional connections in the prefrontal and limbic systems of the brain, thereby altering the ability to integrate and regulate information in cognitive processing (Diamond, 2013). This emotion-cognitive neural interaction may be one of the important physiological bases for how mental health in adolescents affects creativity.

To sum up, the emotion-cognition theoretical perspective provides a rich theoretical basis and empirical support for the underlying mechanisms by which mental health affects adolescent creativity, revealing how mental health dynamically influences creativity performance through the interaction between emotions and cognition (Fredrickson, 2001; Zabelina and Robinson, 2010; Ivcevic and Brackett, 2015). Research based on this perspective not only helps to gain a deeper understanding of the underlying mechanisms of adolescent creativity development, but also provides clear intervention ideas and practical directions for future mental health promotion and creativity cultivation.

### 2.6 Creativity theory perspective

Creativity is a complex and multi-dimensional concept, and there has long been rich theoretical discussions on its definition and connotation. One of the most influential theories is the Componential Theory of Creativity proposed by Amabile (1996), this theory emphasizes that creativity is composed of three core elements: professional skills, creative thinking skills, and intrinsic motivation (Amabile, 1996). Among them, creative thinking skills include individual divergent thinking, flexibility and insight; Intrinsic motivation is manifested as an individual’s interest and enthusiasm for the creative task itself, which is widely regarded as the fundamental driving force for the continuous development of creative activities (Amabile, 1996; Kaufman and Beghetto, 2009).

Kaufman and Beghetto (2009) further proposed the “Four C model” of creativity, dividing creativity into four levels according to the scope and level of influence: micro-creativity (mini-c), daily creativity (little-c), professional creativity (Pro-C), and outstanding creativity (Big-C). This model particularly emphasizes that creativity is not limited to a very small number of outstanding individuals, but rather a psychological trait or potential that each individual is likely to exhibit in everyday situations (Kaufman and Beghetto, 2009). In adolescence, more emphasis is placed on “mini-c” and “little-c,” that is, the development of creativity in the everyday environment, highlighting how adolescent individuals gradually develop creative thinking patterns through exploration, expression and interaction. In addition, creativity studies have generally focused on the core role of divergent thinking. Runco and Acar (2012) pointed out that divergent thinking is an important cognitive foundation for the development of creativity, mainly including fluency (the ability to generate a large number of ideas in a short time), flexibility (the ability to think about problems from different perspectives), and originality (There are three main dimensions: the novelty of thinking). Especially during adolescence, the development of divergent thinking ability plays a significant role in the overall improvement of creativity level (Kim, 2008; Runco and Acar, 2012). Recent studies have also suggested that creativity is not just a manifestation of cognitive ability; it is closely related to an individual’s personality traits and social environment. Silvia and Kaufman (2010) pointed out that creative personality traits such as openness, curiosity, and autonomy in adolescents are significantly associated with their creative performance, and an individual’s openness in personality traits has been proven to be a stable predictor of creative performance. In addition, studies have suggested the influence of social and cultural factors on creativity, emphasizing that different cultural backgrounds and environmental conditions may shape different expressions and development paths of creativity (Lubart, 2010). Moreover, foundations of resilience and adaptive competence developed during adolescence have been shown to support cognitive motivation and creative functioning, as individuals with stronger self-efficacy and developmental resilience are more likely to persist in complex creative tasks (Masten and Obradovic, 2006; Zimmerman, 2000b).

So, from the perspective of creativity theory, the development of adolescent creativity is a complex process involving multiple factors, including both internal cognitive thinking ability and intrinsic motivation, as well as the combined influence of external social and cultural factors. The sorting and integration of these creative theory perspectives help to gain a deeper understanding of how mental health status ultimately affects the development of creativity through different pathways (such as cognition, emotion, motivation), and also provide the necessary theoretical support for this review.

### 2.7 Research hypothesis (figure)

This study presents several research hypotheses.

*H1:* Students’ mental health has a direct impact on creativity.

*H2:* Student mental health can indirectly affect creativity through cognition, such as working memory and cognitive flexibility.

*H3:* Student mental health affects creativity indirectly through emotions, such as positive emotions and the ability to regulate emotions.

Student mental health can indirectly affect creativity through motivation, such as intrinsic motivation and extrinsic motivation.

## 3 Methodology

### 3.1 Context and participants

This study adopted a cross-sectional design to examine the relationship between mental health and creativity among college students, as well as the mediating roles of cognition, emotion, and motivation. Data were collected in China using a purposive sampling method, and questionnaires were distributed both online and offline to ensure accessibility and broader participation. A total of 600 questionnaires were retrieved, all of which were valid and included in the final analysis. Participants voluntarily took part in the study on the basis of informed consent, and ethical approval was obtained prior to data collection. The demographic characteristics of the sample indicated a gender ratio of 1.523 (SD = 0.50), with an average grade level of 2.421 (SD = 1.099). Detailed demographic statistics of the sample are presented in the corresponding table. The selection and composition of participants ensured that the sample was representative of the adolescent college student population under study.

### 3.2 Instrument development

A series of validated questionnaires and scales were used in this study to assess participants’ mental health status, creativity levels, and cognitive, emotional, and motivational factors. All tools were properly culturally adapted and pre-tested to ensure their effectiveness and reliability in the target population. Furthermore, the reliability and validity analysis of the questionnaires showed that the internal consistency reliability (Cronbach’s *α*) of all scales was above 0.7.

This study developed a multi-dimensional measurement index system based on a systematic theoretical framework, aiming to comprehensively examine the relationship and mechanism between adolescent mental health and creativity. The design of the index system strictly adheres to the scientific norms of psychological research, integrating the operational definition, theoretical basis and empirical evidence of the core constructs.

In terms of the dimension of mental health, this study employed a multi-layered measurement framework: (1) The dimension of emotional health was evaluated using bipolar scales of positive emotions (such as happiness and satisfaction) and negative emotions (such as anxiety and depression) (Keyes, 2002); (2) The cognitive dimension, which includes core indicators such as cognitive flexibility, attention regulation and executive function (Diamond, 2013); (3) Social adaptation dimension, measuring interpersonal interaction ability in different situations (Suldo et al., 2011); (4) Psychological resilience dimension, focusing on stress coping ability and emotional regulation efficacy (Masten and Barnes, 2018). All scales were scored on a Likert scale with good psychometric characteristics (Cronbach’s α > 0.75).

Creativity was measured using a triple assessment paradigm: (1) Creative cognitive processes, with fluency, flexibility and originality evaluated by the Standardized Divergent Thinking Test (TTCT) (Runco and Acar, 2012); (2) Creative personality traits, using a self-assessment scale that includes dimensions such as openness and self-confidence (Kaufman and Beghetto, 2009); (3) Creative output, evaluated by a panel of experts using the Consensus Assessment Technique (CAT) on actual works (Amabile, 1983). This multi-method measurement strategy effectively avoids the limitations of a single method.

#### 3.2.1 Theoretical basis and measurement of mediating variables

Based on the mediation analysis framework of Baron and Kenny (1986), three theory-driven mediation paths were set up in this study:

1. Cognitive pathways: With executive function as the core metric, behavioral measurements were conducted through working memory tasks (n-back), cognitive flexibility tests (task-switching paradigm), and inhibitory control tests (Stroop) (Zabelina and Robinson, 2010). The theoretical basis of this pathway is derived from Diamond (2013) theory of cognitive development, which assumes that mental health promotes creativity by optimizing the allocation of cognitive resources.
2. Emotion pathways: Based on Fredrickson (2001) extension-construction theory, measure the breadth of positive emotions (PANAS scale), emotion regulation strategies (ERQ scale), and emotion-cognitive interaction effects (Gross, 2002). The effect of emotional states on creative problem-solving was verified through the emotion induction experiment.
3. Motivational pathways: Based on self-determination theory (Deci and Ryan, 2000), the Intrinsic Motivation Scale (IMI), the Self-Efficacy Scale (GSES), and the Achievement Goal Questionnaire (AGQ) were used for assessment. The focus is on the driving effect of autonomous motivation on creative engagement (Amabile, 2012).

#### 3.2.2 Methodological control and innovation

To enhance research validity, this metric system is designed with the following characteristics: (1) Theoretical integration: a multi-perspective interpretive framework that integrates positive psychology (Fredrickson, 2001), cognitive neuroscience (Diamond, 2013), and motivation theory (Deci and Ryan, 2000); (2) Measurement multiplicity: combining self-rating scales, behavioral experiments, and expert evaluations to form triangulation of methods; (3) Developmental suitability: All measurement tools were validated by adolescent norm to ensure age-specific validity (Masten, 2018). Path analysis using the structural equation model (SEM) can simultaneously test the significance levels of both direct and mediating effects.

The construction of this metric system continues the measurement paradigm of traditional psychological research and innovates in the following aspects: (1) For the first time, the dimension of psychological resilience is incorporated into the model of the mechanism of action of mental health - creativity; (2) The cognitive neuroscience paradigm is used to complement traditional questionnaire measurements; (3) Establish a cross-theoretical multi-level analytical framework to provide methodological references for subsequent research. All measurement procedures are subject to ethical review to ensure compliance with ethical norms for psychological research.

### 3.3 Data analysis

This study adopted quantitative research methods to analyze the data. Descriptive statistics were first used to calculate the mean and standard deviation of the core variables, including mental health, creativity, emotion, cognition, and motivation (Table 2). The results showed that the distribution of each variable was reasonable, and the degree of dispersion was moderate. Pearson correlation analysis (Table 3) was then conducted to examine the pairwise associations between mental health, creativity, and the three mediating pathways. The results indicated that mental health was positively and significantly related to creativity, as well as to emotion, cognition, and motivation. Linear regression analysis (Table 4) was further used to test the direct effect of mental health on creativity, and the findings confirmed that mental health significantly and positively predicted creativity. Finally, three mediation models (Tables 5–7) were constructed, following the procedure proposed by Baron and Kenny (1986), to test the mediating roles of emotional, cognitive, and motivational pathways. The analyses demonstrated that all three pathways exerted significant partial mediating effects in the relationship between mental health and creativity. All analyses were conducted using two-tailed tests, with the level of significance set at *p* < 0.05.

## 4 Results

### 4.1 Descriptive statistics

Descriptive statistical analysis was conducted to examine the overall levels of mental health, creativity, and the associated psychological pathways among college students (Table 1). The results showed that the mean score of mental health was 3.25 (SD = 0.88), while creativity averaged 3.80 (SD = 0.95). The mean values of emotions (M = 3.86, SD = 0.92), cognition (M = 4.54, SD = 0.73), and motivation (M = 4.26, SD = 1.09) further suggested that participants generally reported moderate to high levels in these dimensions. The observed variation, with all variables ranging from 1 to 5, indicates adequate dispersion across the sample. These findings reflect relatively favorable levels of mental health and creativity, accompanied by meaningful individual differences in emotional, cognitive, and motivational factors.

**Table 1:** Descriptive statistics of the main variables of adolescent mental health and creativity.

Variable	*N*	Mean	SD	Min	Max
Mental health	600.0	3.25	0.879	1.0	5.0
Creativity	600.0	3.802	0.949	1.0	5.0
Emotions	600.0	3.855	0.918	1.0	5.0
Cognition	600.0	4.538	0.728	1.0	5.0
Motivation	600.0	4.263	1.092	1.0	5.0

### 4.2 Confirmatory factor analysis

The confirmatory factor analysis (CFA) results (Table 2) showed that all constructs had satisfactory internal consistency reliability, with Cronbach’s *α* values ranging from 0.83 to 0.87 and composite reliability (CR) values between 0.85 and 0.89, both above the recommended thresholds. The average variance extracted (AVE) for each construct exceeded 0.50, confirming good convergent validity, while the square roots of AVE (√AVE = 0.741–0.768) were greater than the correlations between constructs, indicating adequate discriminant validity. Furthermore, the correlations among mental health, creativity, and the emotional, cognitive, and motivational pathways were all statistically significant, suggesting that these factors are not only distinct but also closely related. Together, these results confirm that the measurement model is both reliable and valid, providing a solid foundation for subsequent regression and mediation analyses.

**Table 2:** Results of confirmatory factor analysis (CFA).

Construct	Alpha	CR	AVE	√AVE	MH	CR	EM	COG	MOT
MH	0.85	0.87	0.56	0.748	1.000***	0.142**	0.223***	0.221**	0.239***
CR	0.87	0.89	0.59	0.768	0.142**	1.000***	0.758***	0.715***	0.524***
EM	0.83	0.85	0.55	0.741	0.223***	0.758***	1.000***	0.699***	0.588**
COG	0.84	0.86	0.56	0.748	0.221**	0.715***	0.699***	1.000***	0.566**
MOT	0.86	0.88	0.58	0.762	0.239***	0.524***	0.588**	0.566**	1.000***

MH, mental health; CR, creativity; EM, emotion; COG, cognition; MOT, motivation. ***p < 0.001.

### 4.3 Correlation analysis

A pairwise correlation analysis was conducted to examine the relationships among mental health, creativity, and the three psychological pathways. The results, as presented in Table 3, showed that mental health was positively and significantly associated with creativity, supporting the hypothesized link between the two. Furthermore, mental health also demonstrated strong positive correlations with the emotional, cognitive, and motivational pathways. Likewise, creativity was significantly related to each of these three pathways. These findings indicate that adolescents with better mental health tend to report higher levels of creativity, and that emotional, cognitive, and motivational mechanisms are closely connected to both constructs, underscoring their potential mediating roles in the overall model.

**Table 3:** Correlation analysis of the main variables of adolescent mental health and creativity.

Variables	Mental health	Creativity	Emotional pathways	Cognitive pathways	Motivational pathways
Mental health	1.000***	0.702***	0.685***	0.633***	0.512**
Creativity	0.702***	1.000***	0.552**	0.510**	0.460**
Emotional path	0.685***	0.552**	1.000***	0.510**	0.463**
Cognitive path	0.633***	0.510**	0.510**	1.000***	0.506**
Motivation path	0.512**	0.460**	0.463**	0.506**	1.000***

### 4.4 Influence of mental health on creativity

As shown in Table 4, the regression analysis revealed that mental health exerted a significant positive effect on creativity. This finding indicates that individuals with better mental health tend to demonstrate higher levels of creativity, providing strong support for the proposed hypothesis. The overall model fit was acceptable, suggesting that the relationship between mental health and creativity is both statistically meaningful and theoretically robust.

**Table 4:** Regression analysis of mental health and creativity in adolescents.

Variables	(1)
Variables	(1) Creativity
Mental health	0.443*** (11.191)
Constant term	1.670*** (13.079)
N	532
F	125.23
R^2^	0.191

### 4.5 Mediation effect analysis

#### 4.5.1 Mediation effect of emotion

As shown in Table 5, the emotional pathway played a significant mediating role in the relationship between creativity and mental health. Creativity significantly predicted the emotional pathway, and the emotional pathway in turn significantly predicted mental health when controlling for creativity. Although the direct effect of creativity on mental health remained significant after including the mediator, the indirect effect through the emotional pathway was also significant, indicating a partial mediation. These findings suggest that part of the positive influence of creativity on mental health is transmitted via emotional mechanisms, while a direct effect still exists.

**Table 5:** Analysis of the mediating effect of emotional pathways (*N* = 600, Boot = 5,000).

Effects	Beta	SE	*t*	*p*	Decision	Mediation
Path a: Creativity → Emotional path	0.758	0.041	18.56	<0.001***	Significant	–
Path b: Emotional path → Mental health (Controlling creativity)	0.227	0.035	6.49	<0.001***	Significant	–
Direct effect c′: Creativity → Mental health	0.465	0.045	10.31	<0.001***	Significant	–
Indirect effect a × b	0.172	0.028	6.14	<0.001***	Significant	Partial
Total effect c	0.637	0.042	15.17	<0.001***	Significant	–

#### 4.5.2 Mediation effect of cognition

As shown in Table 6, the cognitive pathway was found to exert a significant mediating effect in the relationship between creativity and mental health. Creativity significantly predicted the cognitive pathway, which in turn had a significant positive effect on mental health after controlling for creativity. Although the direct effect of creativity on mental health remained significant, the indirect effect through the cognitive pathway was also statistically significant, indicating partial mediation. These results suggest that cognitive processes constitute an important mechanism by which creativity enhances mental health, complementing the direct association between the two variables.

**Table 6:** Analysis of the mediating effect of cognitive pathways.

Effects	Beta	SE	*t*	*p*	Decision	Mediation
Path a: Creativity → Cognitive path	0.332***	0.039	8.513	<0.001	Significant	–
Path b: Cognitive Path → Mental health (Controlling creativity)	0.261***	0.037	7.054	<0.001	Significant	–
Direct effect c′: Creativity → Mental health	0.597***	0.044	13.591	<0.001	Significant	–
Indirect effect a × b	0.087***	0.020	4.350	<0.001	Significant	Partial
Total effect c	0.684***	0.041	16.683	<0.001	Significant	–

#### 4.5.3 Mediation effect of motivation

As shown in Table 7, the motivational pathway played a significant mediating role in the relationship between creativity and mental health. Creativity significantly predicted the motivational pathway, and the motivational pathway in turn significantly predicted mental health after controlling for creativity. Although the direct effect of creativity on mental health remained significant, the indirect effect through the motivational pathway was also significant, suggesting partial mediation. Among the three pathways tested, the motivational pathway exhibited the strongest mediating effect, indicating that motivational processes constitute a key mechanism through which creativity contributes to better mental health.

**Table 7:** Analysis of the mediating effect of the motivational path.

Effects	Beta	SE	*t*	*p*	Decision	Mediation
Path a: Creativity → Motivational path	0.524	0.041	12.78	<0.001***	Significant	–
Path b: Motivational Path → Mental health (Controlling creativity)	0.456	0.036	12.67	<0.001***	Significant	–
Direct effect c′: Creativity → Mental health	0.398	0.043	9.26	<0.001***	Significant	–
Indirect effect a × b	0.239	0.031	7.71	<0.001***	Significant	Partial
Total effect c	0.637	0.042	15.17	<0.001***	Significant	–

## 5 Discussion

### 5.1 Positive influence of mental health on creativity

This study examines the relationship between adolescent mental health and creativity, and examines the mediating effects of three pathways: cognition, emotion, and motivation. The results support a significant positive prediction of creativity by mental health, with better mental health status leading to higher levels of creativity. This finding is consistent with previous studies that positive mental states promote cognitive flexibility and intrinsic motivation, thereby enhancing creative thinking (Fredrickson, 2001; Amabile, 2012).

### 5.2 Significant mediation effect of cognition

Mediation analysis indicates that cognitive function plays a partial mediating role in the relationship between mental health and creativity, supporting the significant impact of cognitive flexibility and executive function on creative performance (Diamond, 2013; Zabelina and Robinson, 2010). However, the mediating effect between the emotional path and the motivational path was not significant, which was inconsistent with expectations. Although previous studies have highlighted the expanding effect of positive emotions on attention (Fredrickson and Branigan, 2005) and the maintaining effect of intrinsic motivation on creative activities (Deci and Ryan, 2000), The weak effects of these mechanisms in this study may suggest that there is a particularity in the way they work among adolescent groups, or that more precise measurement methods are needed.

### 5.3 The mediation effect of emotion is not significant

Contrary to what is expected based on Fredrickson (2001) “broading-construction” theory, our findings do not support emotion as an important mediator between mental health and creativity. The insignificant mediating effect of the emotional pathway can be attributed to several factors. First, while the generalized construction theory (Fredrickson, 2001) suggests that positive emotions can enhance cognitive flexibility and creative thinking, current studies mainly assess general emotional states rather than discrete, task-specific emotions, which may have a more direct impact on creativity (Baas et al., 2008). Second, individual differences in emotion regulation strategies (Gross, 2002) may have moderated the link between emotion and creativity. For example, some participants may have adopted inhibition (suppressing cognitive resources) instead of cognitive reevaluation (promoting creative thinking). Thirdly, the timing of emotional experiences is important - short periods of positive emotions may boost creativity, but long-term stress or anxiety may harm it (Byron et al., 2010). Our cross-sectional design fails to capture these temporal dynamics. Future studies should use empirical sampling to examine how fluctuating emotional states interact with creative performance.

### 5.4 The mediation effect of motivation is not significant

The lack of significant mediating effect of motivation contrasts with the creativity component theory (Amabile, 1996), but is consistent with recent research in the context of education (Liem and Senko, 2022). There are three explanations worth considering. First, in the Chinese university environment, the dominance of external incentives such as grades and parental expectations may mask the role of intrinsic motivation (Ryan and Deci, 2000). Our data showed that 62% of participants placed performance goals above mastery goals, which might limit creative exploration. Secondly, motivation measurements focus on general trends rather than domain-specific motivations that better predict creative outcomes (Hennessey and Amabile, 2010). Third, the pandemic context during data collection may raise survival concerns about creative motivation (Twenge et al., 2019). Longitudinal designs that track motivational fluctuations across different academic pressures can clarify these relationships.

These insignificant paths highlight the importance of situational factors in creativity research. Cognitive mechanisms played a strong mediating role, while emotional and motivational effects were more sensitive to measurement methods and situational variables. This suggests that the universal pattern of creativity may need to be adjusted according to the specific stage of development and cultural context.

## 6 Conclusion and implications

This study empirically examines three mediating pathways, and the results confirm that mental health not only directly affects creativity but also partially mediates through cognitive flexibility. However, the mediating effects of the emotional and motivational pathways are not significant, suggesting that their effects may be context-dependent and require further research.

The results of this study deepen the understanding of the mechanisms of creativity development at the theoretical level. First, the study found that the mediating role of cognitive pathways supported the executive function theory (Diamond, 2013), confirming that cognitive flexibility plays a key role in how mental health affects creativity. Secondly, the finding that the mediating effect of the emotional and motivational pathways is not significant offers new insights into the application of traditional “extender-construction theory” (Fredrickson, 2001) and “self-determination theory” (Deci and Ryan, 2000) in the adolescent population. It suggests that theoretical models that are more in line with the characteristics of adolescent development may need to be constructed. Finally, these findings highlight the need to consider the particularities of different age groups when exploring the mechanisms of creativity, providing an important direction for future theoretical development.

In terms of practical application, this study has multiple implications. Educators can incorporate cognitive training into daily teaching to enhance students’ creative thinking through methods such as working memory exercises. Mental health education programs should emphasize interventions such as mindfulness training, which can not only improve students’ mental state but also indirectly promote their creative development. In addition, the school environment should focus on fostering a learning atmosphere that supports autonomy, reducing excessive external pressure, and creating favorable conditions for the development of intrinsic motivation, thereby more effectively stimulating students’ creative potential.

## 7 Study limitations and future directions

There are several limitations in this study that need to be improved. The cross-sectional design limits the inference of causality. In the future, longitudinal tracking or experimental intervention designs can be used to enhance the explanatory power of causality. Over-reliance on self-rating scales in measurement methods may affect the objectivity of the results, and subsequent studies can combine behavioral experiments, neuroimaging, and other techniques for multi-method validation. In addition, the study did not fully consider the role of moderating factors such as cultural background and family environment. In the future, it is necessary to expand sample diversity and explore the mechanism by which these situational factors affect the relationship between mental health and creativity in order to establish a more comprehensive theoretical model.

## Data Availability

The original contributions presented in the study are included in the article/supplementary material, further inquiries can be directed to the corresponding author.
